# Supplementary material for: Cytotoxicity and Biomineralization Potential of Flavonoids Incorporated into PNVCL Hydrogels
Source: J Funct Biomater. 2023 Mar 2;14(3):139. doi: 10.3390/jfb14030139 (PMC10058549; doi:10.3390/jfb14030139)
Supplement: Supplementary file 1 [file jfb-14-00139-s001.zip › jfb-2207936-supplementary.pdf]

Supplementary Materials

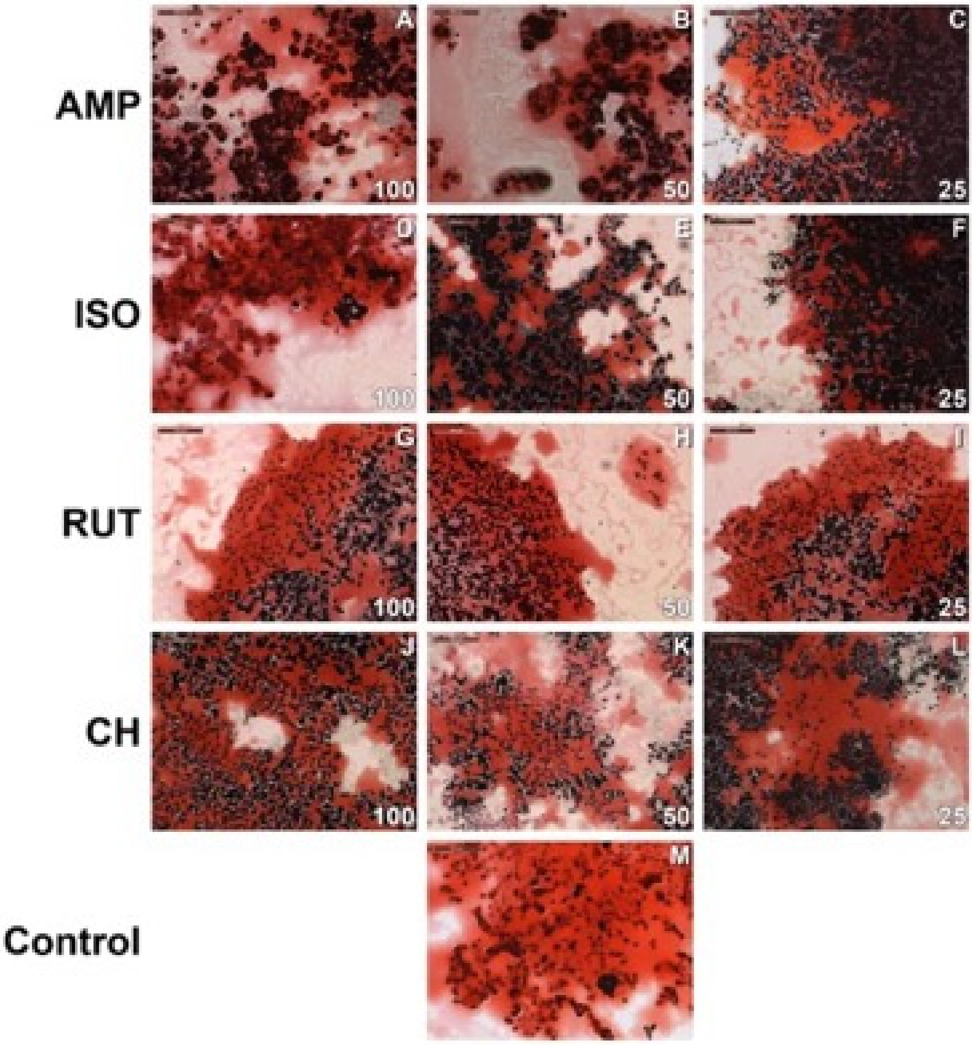

Figure S1. Representative images of alizarin red staining obtained by inverted light microscope, indicating the biomineralizing effect of flavonoids on the MDPC-23 cells. All groups induced high nodules deposition, varying according to their concentration. AMP at 10 μM (A), AMP at 50 μM (B), AMP at 25 μM (C), ISO at 100 μM (D), ISO at 50 μM (E), ISO at25 μM (F), RUT at 100 μM (G), RUT at 50 μM (H), RUT at 25 μM (I) and CH at 100 μM (J), CH at 50 μM (K), CH at 25 μM (L) Control DMEM (M). 4× magnification.

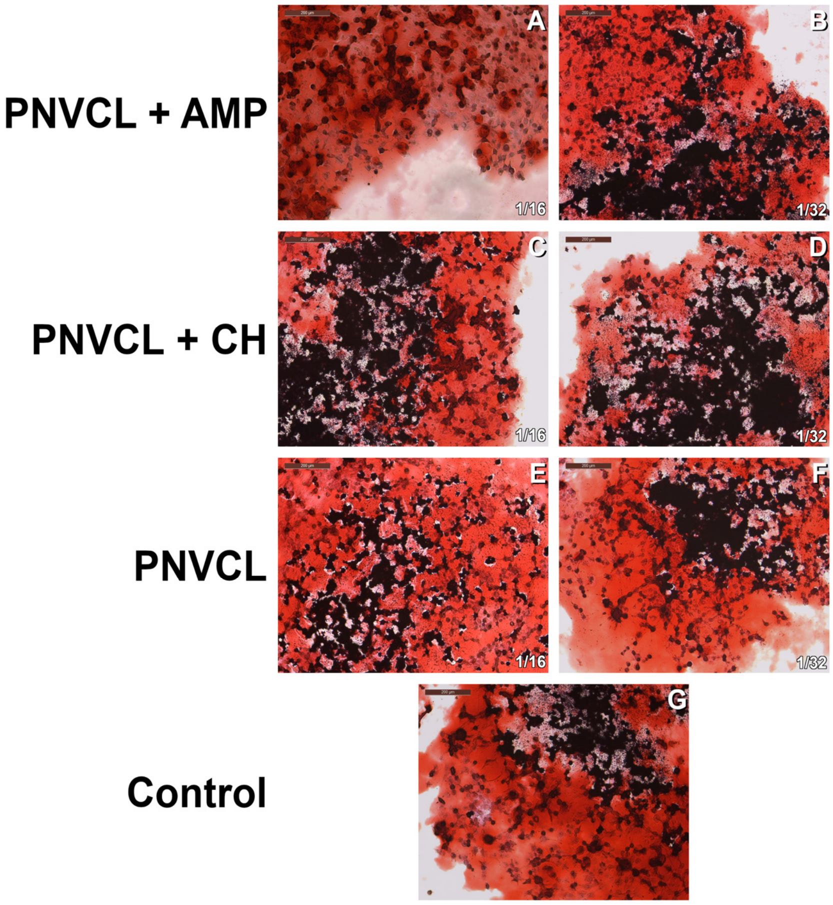

Figure S2. Images of alizarin red staining obtained by inverted light microscope, indicating the biomineralizing effect of 48 h hydrogels extracts on the MDPC-23 cells. The greatest nodules deposition can be seen in the groups PNVCL+AMP 1/32 and both dilutions of PNVCL+CH (1/16 and 1/32).PNVCL+AMP 1/16 (A), PNVCL+AMP 1/32 (B), PNVCL+CH 1/16 (C), PNVCL+CH 1/32 (D), PNCVL 1/16 (E), PNVCL 1/32 (F) and control DMEM (G). 4× magnification.

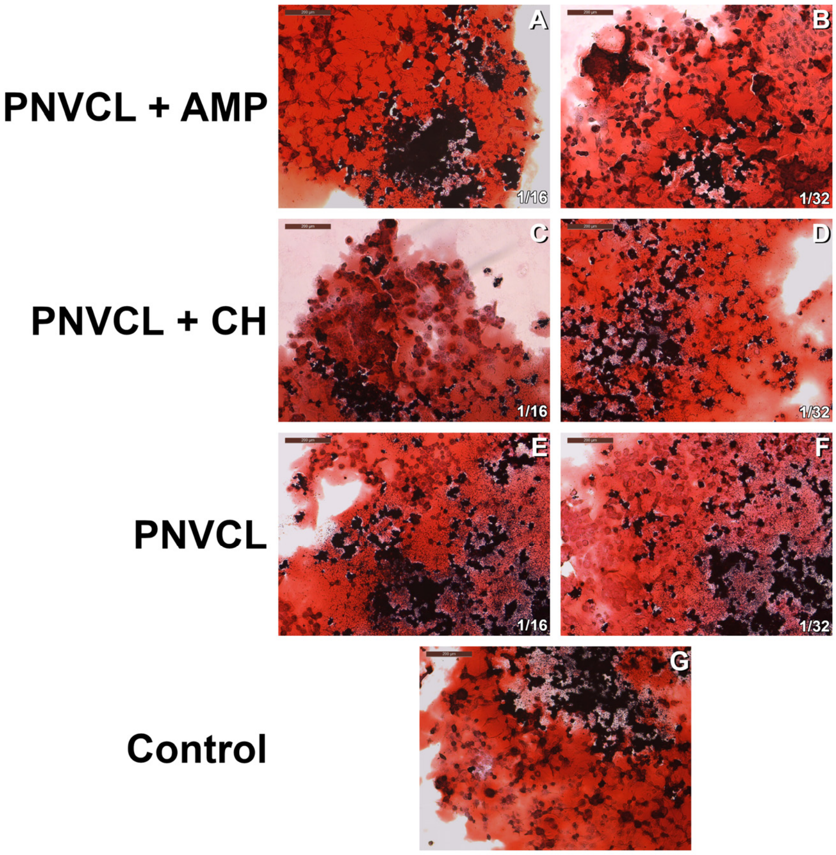

Figure S3. Images of alizarin red staining obtained by inverted light microscope, indicating the bioactive effect of 7 days hydrogels extracts on the MDPC-23 cells relative to mineralized nodules formations. The greatest nodules deposition can be seen in the groups PNVCL + AMP and PNVCL + CH , both at 1/16 and 1/32 dilutions. PNVCL+AMP 1/16 (A), PNVCL+AMP 1/32 (B), PNVCL+CH 1/16 (C), PNVCL+CH 1/32 (D), PNCVL 1/16 (E), PNVCL 1/32 (F) and control DMEM (G). 4× magnification.
